# Case report: Application of metagenomic next-generation sequencing in the diagnosis of visceral leishmaniasis and its treatment evaluation

**DOI:** 10.3389/fmed.2022.1044043

**Published:** 2023-01-13

**Authors:** Qiuping Liang, Xiaogong Liang, Dengwei Hong, Yuan Fang, Lanlan Tang, Jiao Mu, Xiaoli Tan, Feng Chen

**Affiliations:** ^1^Department of Respiratory and Critical Care Medicine, The Third People's Hospital of Chengdu, Chengdu, China; ^2^Department of Hematopathology, Mianyang Central Hospital, Mianyang, China; ^3^Genoxor Medical Science and Technology Inc., Shanghai, China

**Keywords:** visceral leishmaniasis, mNGS, *Leishmania*, treatment efficacy, case

## Abstract

Visceral leishmaniasis is a vector-borne infection by the *Leishmania* spp., a parasite. Although the overall incidence of visceral leishmaniasis is low, the disease still occurs frequently in some high-risk areas. In our study, two patients were admitted to the hospital with an unprovoked and recurrent high fever, and the condition was not improved after antibiotics administration. Meanwhile, bone marrow aspiration smears failed to find out any pathogen. Finally, *Leishmania*-specific nucleic acid sequences were successfully detected in the peripheral blood of two patients through metagenomic next-generation sequencing (mNGS), which was further confirmed by bone marrow smear microscopy and antibody tests. After targeted treatment for visceral leishmaniasis in the patients, mNGS reported a decrease in the reads number of *Leishmania* sequence. The results indicate the feasibility of mNGS in detecting *Leishmania* spp. in peripheral blood samples. Its therapeutic effect evaluation may be achieved through a comparative analysis of the number of reads before and after the treatment.

## Introduction

Leishmaniasis is a vector-borne infection caused by protozoan parasites of the genus *Leishmania*, and is transmitted through the bite of female *Phlebotomus Sandflies* (1–3). There are several forms of leishmaniasis in China, wherein visceral leishmaniasis cases predominate, whose main causative agents are *Leishmania donovani* (*L. donovani*) and *Leishmania infantum* (4). According to the 2011 Chinese official record, the annual incidence of visceral leishmaniasis is very low, i.e., only 0.03/100,000, with an overwhelming majority of cases reported from sites of endemicity in the western and northwestern regions of China (5). Due to the low incidence, visceral leishmaniasis is among the most neglected infectious diseases. However, it is also the most severe form, with a poor prognosis and a high fatality rate. So far, early diagnosis and treatment are essential in controlling visceral leishmaniasis.

Due to the low incidence of visceral leishmaniasis and atypical symptoms in some patients, its diagnostic procedure is usually not straightforward. It is generally made by combining clinical signs with parasitological and serological tests. Chronic fever, hepatosplenomegaly, and pancytopenia are the primary classical manifestations of visceral leishmaniasis (6), varying from the host's immune status, the parasite, and immunoinflammatory responses (7). Microscopic examination of aspirate smears in bone marrow, lymph nodes, and spleen is the most reliable diagnostic method for visceral leishmaniasis. In general, splenic aspirate shows the highest diagnostic value (with specificity and sensitivity of more than 90%), followed by bone marrow (with sensitivity in the range of 53 and 86%) and lymph nodes (with sensitivity ranging from 53 to 65%) (8). The low sensitivity of bone marrow cytomorphology limits its application in accurately identifying or excluding suspicious patients. The serological examination based on the rK39 antigen is the most rapid and widely used in China. However, specific antibody testing may not be arranged timely for patients with atypical symptoms due to the low incidence of visceral leishmaniasis.

Recently, next-generation sequencing (NGS) technology has been applied to the etiological diagnosis of infectious diseases, known as metagenomic next-generation sequencing (mNGS) (9). Due to its unbiased and hypothesis-free characteristics, mNGS has emerged as a vital method in identifying complex microorganisms to culture and diagnosing infectious diseases with low incidence. As recently reported, mNGS allows for *Leishmania* detection and early diagnosis of visceral leishmaniasis, improving the prognosis of the patients (10). In this case report, two patients with fever of unknown origin were enrolled, with some abnormal items in their physical examinations, including blood biochemistry, liver function, and blood routine. However, bone marrow aspiration smears failed to find any pathogen until *mNGS identified Leishmania* in the peripheral blood samples of these two patients. Then, *Leishmania* was found in the bone marrow aspiration smears and further confirmed with the antibody tests. Visceral leishmaniasis was diagnosed, and antimonials were used as the conventional therapy. After the symptoms disappeared, mNGS was performed again to assess the treatment effect by comparing the number of reads before and after the treatment. The detailed histories of both patients were as follows.

## Case description

### Case 1:

On January 9, 2021, a 52-year-old female patient who lives in Jiuzhaigou in the Sichuan province of China was admitted to another hospital because of an unprovoked high fever (Table 1). The body temperature reached 39.6°C, accompanied by coughing, sputum expectoration, tiredness, anhelation, chilly, and shivering. A mass of rash was seen on the face, chest, and groin. She was checked by ultrasound in urinary system, brain magnetic resonance imaging (MRI) scan, and chest computed tomography (CT), but no significant abnormality was seen. Based on the above symptoms, antibiotics ceftriaxone sodium and clindamycin were used for anti-infection, and methylprednisolone was administered as an anti-asthmatic drug.

**Table 1:** Demographics and clinical presentations of the 2 cases.

**Items**	**Case 1**	**Case 2**
Gender	Female	Male
Age, y	52	55
Past medical history	Anemia, history of blood transfusion	Pancreatitis, septicopyemia
Occupation	Unemployed	Farmer
Family history	Thalassemia	None
History of the epidemic area	Sichuan	Sichuan, Xinjiang
Chief complaints	Recurrent fever for 7 d, up to 39.6°C	Recurrent fever for 1 m, up to 41°C
Accompanying symptoms	Cough, expectoration, fatigue, anhelation, chilly, shiver	Shiver
Clinical signs	Hepatosplenomegaly, pancytopenia	Hepatosplenomegaly

On January 11, 2021, the concentration of folic acid (FOL), vitamin B12 (VB12), and serum ferritin (SF) was determined, with the level of SF on the high side. On January 13, 2021, the bone marrow aspiration smear showed a low proliferation rate of nucleated cells, a downside of granulocyte/erythrocyte ratio, and some erythroblasts presented with abnormal morphology. In addition, a significant reduction of iron content in the bone marrow was seen, and the platelet production by megakaryocytes was poor. Scattered and clustered platelets could be seen in the bone marrow. After administering drugs for a week, she recovered from symptoms like coughing, sputum expectoration, tiredness, and shortness of breath. However, the fever was not on the mend. On January 14, 2021, the results of the blood routine indicated decreased levels of white blood cells (WBC), red blood cells (RBC), hemoglobin (HGB), and platelet (PLT). The timeline of diagnosis and treatment of case 1 is demonstrated in Table 2.

**Table 2:** Timeline of diagnosis and treatment in the case 1.

**Timeline**	**Physical examination items**	**Medical results**	**Diagnosis and treatment**
2021-1-9	Urinary ultrasound Brain MRI scan Chest CT	Urate crystal in bilateral renal Slight ischemic lacuna in the brain No abnormality seen	Treatment: Ceftriaxone sodium, clindamycin, methylprednisolone
2021-1-11	Anemia detection	FOL 16.62 ng/ml VB12 343.7 ng/ml SF 614.8 ng/ml	
2021-1-13	Bone marrow aspiration smear 24 h urine protein quantitation Blood clotting assays	Low proliferation rate of nucleated cells 563.8 mg/24 h Fibrinogen 4.59 g/L D-dimer 3.86 mg/L	
2021-1-14	Blood routine	WBC 3.3 × 10^9^/L RBC 2.88 × 10^12^/L HGB 63 g/L PLT 50 × 10^9^/L	
2021-1-15	Urine routine Blood routine CRP PCT IL-6 Blood pathogens detection Respiratory tract pathogens detection Coombs test Polyspecific antibody Chest CT scan Abdominal B-mode ultrasound	Occult blood 10 (±) RBC/ul WBC 3.43 × 10^9^/L HGB 66 g/L PLT 61 × 10^9^/L ESR 99 mm/H 106.96 mg/L 0.41 ng/ml 164.3 pg/ml Negative Weakly positive Parainfluenza Virus Types 1-2-3 IgM antibody Positive Positive Splenomegaly Hepatosplenomegaly	Tentative diagnosis: Fever, with a moderate anemia, chronic nephritis, hypertension of grade 2, and type 2 diabetes Treatment: Tazocin, moxifloxacin, oseltamivir, FOL Tablets, VB12, clexane
2021-1-19	Bone marrow aspiration smear	Active bone marrow hyperplasia, dysplastic changes in erythroid cells	
2021-1-20	Bone marrow chromosome karyotyping	No abnormality seen	
2021-1-21	Analysis of gene mutation in myeloid blood diseases	No pathogenic mutation found	
2021-1-24	Peripheral blood mNGS detection	*Leishmania* genus-specific sequence	
2021-1-26	rK39 test	Positive	Diagnose: Visceral leishmaniasis Treatment: Sodium stibogluconate regimen for 14 days
2021-3-2	Abdominal color ultrasound Blood routine	Splenomegaly WBC 4.22 × 10^9^/L HGB 75g/L PLT 136 × 10^9^/L	
2021-3-8	Peripheral blood mNGS detection	*Leishmania* genus in mcfDNA	

MRI, magnetic resonance imaging; CT, computed tomography; FOL, folic acid; VB12, vitamin B12; SF, serum ferritin; mNGS, metagenomic next-generation sequencing; WBC white blood cell; RBC, red blood cell; HGB, hemoglobin; PLT, platelet; CRP C-reactive protein; PCT, procalcitonin; IL-6, interleukin-6; ESR, erythrocyte sedimentation rate.

On January 15, 2021, she was admitted to our hospital (The Third People's Hospital of Chengdu) and confirmed a history of hypertension, diabetes, and anemia, together with thalassemia in her daughter. Based on the medical history and physical examination results, the patient was tentatively diagnosed with “a fever pending for diagnosis, with moderate anemia, chronic nephritis, hypertension of grade 2, and type 2 diabetes.” Chest CT showed splenomegaly, and the abdominal B-mode ultrasound revealed hepatosplenomegaly. After admission, the patient was administered piperacillin sodium/tazobactam sodium and moxifloxacin for anti-infection, oseltamivir for anti-influenza, folic acid tablets, and vitamin B12 as the hematopoietic raw materials, and clexane for preventive anticoagulant.

On January 19, 2021, the bone marrow aspiration smear showed active bone marrow hyperplasia and some dysplastic changes (>10%) in erythroid cells. The patient's fever had not been remitted. On January 20 and 21, no significant abnormality was seen in the bone marrow chromosome karyotyping and gene mutation analysis in myeloid blood diseases.

**Figure 1:**
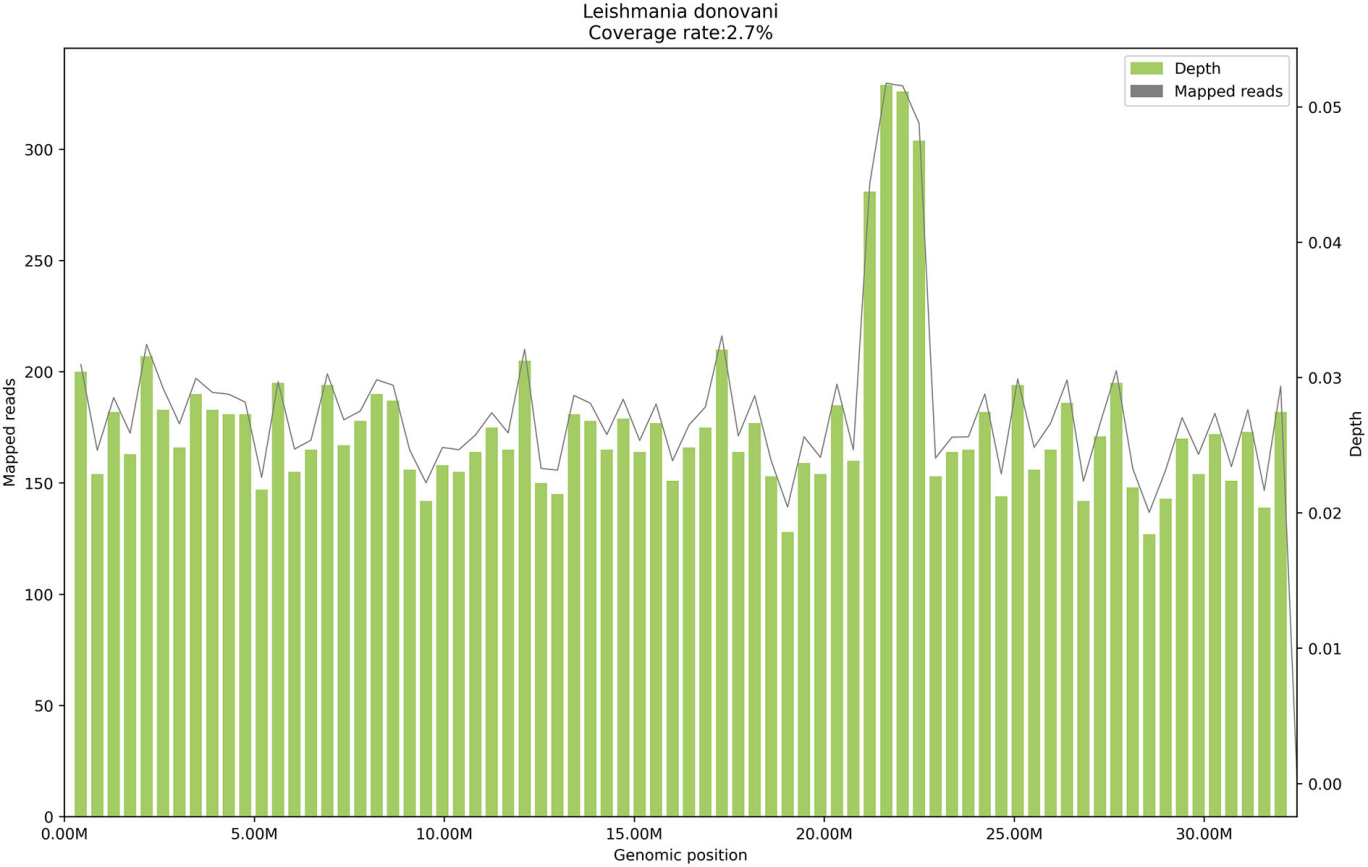
The genome coverage map of *Leishmania donovani* detected by mNGS in case 1. The genome coverage of *Leishmania donovani* in the peripheral blood of case 1 is 2.7%.

On January 24, 2021, peripheral blood mNGS was performed, and 11,552 reads of *Leishmania* genus-specific sequence were detected in microbial cell-free deoxyribonucleic acid (mcfDNA), and 7,509 reads were detected in microbial cell-free ribonucleic acid (mcfRNA) (Table 3), with a genome coverage of 2.7% (Figure 1). The sequencing data were deposited in the database under the Sequence Read Archive (SRA) accession number PRJNA850821. On January 26, the RK39 antibody test showed a positive result. After repeated examinations on the bone marrow aspiration smears, *Leishmania* was found (Supplementary Figure 1). Finally, the patient was diagnosed with visceral leishmaniasis, sepsis, moderate anemia, rash, leukopenia, thrombocytopenia, grade 2 hypertension, and type 2 diabetes mellitus.

**Table 3:** Data and information of mNGS detection.

**Cases**	**Date**	**Test items**	**Total reads number**	**Non-human reads number**	**Leishmania sequence reads**		**Relative abundance**
					**Genus**	**Species**	
Case 1	2021-1-24	CfDNA	19,608,044	177,919	11,552	/	100%
	2021-1-24	CfRNA	31,691,424	433,348	7,509	/	100%
	2021-3-8	CfDNA	33,464,222	284,827	73	/	100%
	2021-3-8	CfRNA	24,055,354	206,251	0	/	100%
Case 2	2021-2-9	CfDNA	20,469,766	848,480	606	15	100%
	2021-3-7	CfDNA	55,851,536	563,416	8	8	4.54%
	2021-3-14	CfDNA	24,341,788	161,652	0	0	/
	2021-3-14	CfRNA	19,744,882	129,835	0	0	/

CfDNA, cell-free deoxyribonucleic acid; CfRNA, cell-free ribonucleic acid.

On January 26, 2021, a sodium stibogluconate regimen (6 ml/vial, course of treatment for 14 days, D1 3 ml antimonials & 7 ml glucose and sodium chloride injection iv, D2–D14 6 ml antimonials & 4 ml glucose and sodium chloride injection iv) was administrated, and her fever was remitted on that day. On March 2, 2021, the decreased WBC had been resolved, but splenomegaly remained. On March 7, the peripheral blood was collected again for mNGS detection, and 73 reads of the *leishmania-*specific genus sequence were detected in mcfDNA, but none was seen in mcfRNA (Table 3). Meanwhile, the rash on the face, chest, and groin disappeared utterly.

### Case 2:

On January 25, 2021, a 55-year-old man was admitted to our hospital (Mianyang Central Hospital) with recurrent fever, a body temperature of 39.6°C, and acute febrile symptoms. One month before admission, the patient developed a fever of unknown origin, with the highest body temperature of 41°C, accompanied by a shiver. Before admission, he had been treated with piperacillin sodium/tazobactam in the other hospital, but without any improvement. Upon entry, he told a history of pancreatitis and septicemia. He denied any family history. On January 25, 2021, the results of the blood routine revealed reduced levels of WBC, RBC, HGB, and PLT. Hepatosplenomegaly was observed by using the abdominal color ultrasound. Increased amylase and lipase levels were noticed, which are the markers of pancreatitis. A positive result was noted in the Epstein-Barr virus (EBV), but no other blood or respiratory tract pathogen was detected. The timeline of diagnosis and treatment of case 2 is shown in Table 4.

**Table 4:** Timeline of diagnosis and treatment in the case 2.

**Timeline**	**Physical examination items**	**Medical results**	**Diagnosis and treatment**
2021-1-25	Blood routine Abdominal color ultrasound ESR PCT Amylase Lipase Blood pathogens detection Respiratory tract pathogens EBV Tumor marker Blood culture	WBC 2.94 × 10^9^/L RBC 2.68 × 10^12^/L HGB 80 g/L PLT 209 × 10^9^/L Hepatosplenomegaly 24 mm/H 0.499 μg/L 149 U/L 210 U/L Negative Negative Positive CA125 36.92 U/mL CA19-9 35.67 U/mL NSE 13.02 μg/L Negative	Tentative diagnosis: Fever, suspected infection or hemophagocytic syndrome
2021-1-27	Blood routine PCT	WBC 2.24 × 10^9^/L RBC 2.49 × 10^12^/L HGB 75 g/L PLT 112 × 10^9^/L 4.61 μg/L	
2021-1-30	Blood routine PCT	WBC 3.03 × 10^9^/L RBC 2.87 × 10^12^/L HGB 87 g/L PLT 102 × 10^9^/L 1.05 μg/L	
	Amylase Lipase	139 U/L 215 U/L	
	FOL VB12 SF	4.4 ng/ml 439 ng/ml >40,000 ng/ml	
2021-2-4	Blood routine Amylase Lipase	WBC 1.76 × 10^9^/L RBC 2.6 × 10^12^/L HGB 78 g/L PLT 84 × 10^9^/L 189 U/L 182 U/L	
2021-2-6	Blood routine PCT Amylase Lipase SF	WBC 5.78 × 10^9^/L RBC 2.5 × 10^12^/L HGB 75 g/L PLT 78 × 10^9^/L 0.186 μg/L 169 U/L 222 U/L >40,000 ng/ml	
2021-2-7	Bone marrow aspiration smear	Active bone marrow hyperplasia, hemophagocytosis	
2021-2-9	Peripheral blood mNGS detection	*Leishmania donovani*	
2021-2-10	Bone marrow aspiration smear rK39 test Blood routine PCT	Suspected *Leishmania* Positive WBC 1.8 × 10^9^/L RBC 2.4 × 10^12^/L HGB 71 g/L PLT 85 × 10^9^/L 0.184 μg/L	Diagnosis: Visceral leishmaniasis, hemophagocytic syndrome Treatment: sodium stibogluconate for 3 weeks
2021-2-25	Analysis of gene mutation in hemophagocytic syndrome	No pathogenic mutation found	
2021-3-5	Blood routine	WBC 3.65 × 10^9^/L RBC 2.89 × 10^12^/L HGB 95 g/L PLT 240 × 10^9^/L	
2021-3-7	Peripheral blood mNGS detection	*Leishmania*	
2021-3-12	Blood routine SF	WBC 5.97 × 10^9^/L RBC 3.2 × 10^12^/L HGB 104 g/L PLT 342 × 10^9^/L 2,756.38 ng/ml	
2021-3-14	Peripheral blood mNGS detection	None	

EBV, Epstein-Barr virus.

**Figure 2:**
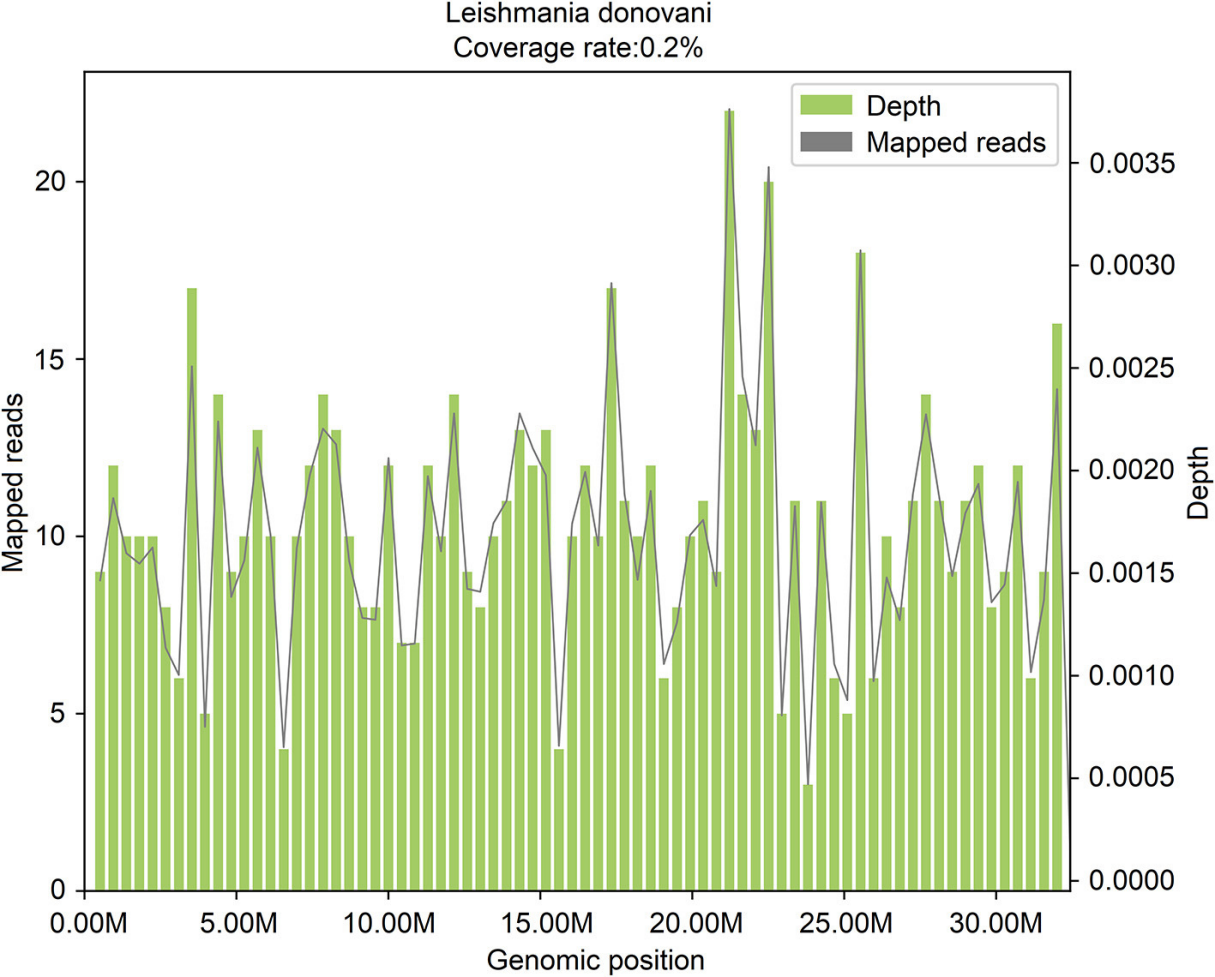
The genome coverage map of *Leishmania donovani* detected by mNGS in case 2. The genome coverage of *Leishmania donovani* in the peripheral blood of case 2 is 0.2%.

In the following 14 days after the admission (from January 25, 2021, to February 6, 2021), blood routine examinations were performed, and the low levels of WBC, RBC, HGB, and PLT were all continued. On February 7, 2021, active bone marrow hyperplasia and hemophagocytosis were seen in bone marrow aspiration smears, but no pathogenic microorganism was found. On February 9, 2021, peripheral blood mNGS was performed, and 606 reads of *Leishmania* genus-specific sequence and 15 reads of *L. donovani*-specific sequence were detected in mcfDNA, accounting for 0.2% of genome coverage (Figure 2). The sequencing data were deposited in the database under the SRA accession number PRJNA850821.

On February 10, 2021, the bone marrow aspiration smear was re-examined, and ten suspected *Leishmania* were seen (Supplementary Figure 2). On the same day, a positive outcome of the rK39 test confirmed *Leishmania*'s existence. The final diagnosis was visceral leishmaniasis, hemophagocytic syndrome, sepsis, liver dysfunction, electrolyte imbalance, hypoproteinemia, coagulopathy, moderate anemia, thrombocytopenia, and leukopenia. Anti-leishmaniasis treatment with sodium stibogluconate (0.6 g, im, qd, course of treatment for 3 weeks) was adopted, and the fever was entirely resolved. On February 25, no *Leishmania* was found in the bone marrow aspiration smear. On February 25, 2021, the results of the analysis on gene mutation in hemophagocytic syndrome manifested no disease-causing mutation, suggesting that this disease is not present in genetic forms in the patient. On March 12, 2021, the WBC count returned to normal, and the levels of HGB and PLT were primarily increased. On March 14, 2021, peripheral blood was collected again for mNGS detection, and no *Leishmania* genus-specific or *L. donovani*-specific sequence was detected in the mcfDNA nor mcfRNA.

## Discussion

Visceral leishmaniasis is an endemic disease, and a history of residence or stays in endemic areas is an essential criterion in its diagnosis (5). The statistical data surveyed from 2004 to 2019 and released by the Chinese Center for Disease Control and Prevention indicated that visceral leishmaniasis is mainly prevalent in five provinces in China, including Xinjiang (2,351 cases), Gansu (1,607 cases), Sichuan (594 cases), Shaanxi (139 cases), and Shanxi (142 cases). These cases account for 96.89% (4,833/4,988) of the nationally reported cases during the same period. In this study, case 1 is a native of Sichuan province and claimed no ecdemic travel history, and can be identified as a local case in Sichuan. For case 2, the source of infection is unclear because he lived in Sichuan province and had a history of Xinjiang residence before the onset of symptoms, which are all endemic areas of visceral leishmaniasis in China (11). This two-case report highlights the necessity to strengthen the surveillance and control of this infectious disease in its epidemic areas, despite its low incidence in China.

Owing to the wide range of non-specific clinical symptoms, patients with leishmaniasis frequently could not be diagnosed using conventional methods. For example, in seven patients whose blood mNGS findings pointed to *Leishmania* infection and were finally diagnosed with leishmaniasis, only three individuals tested positive for rK39, and two had *Leishmania* amastigotes identified in their bone marrow (12). In the two cases of this study, bone marrow aspirate smears failed to find *Leishmania* before mNGS detection, because of short of knowledge on this parasite and experience of visceral leishmaniasis diagnosis, especially in the local hospital. Due to a history of anemia in case 1, together with the thalassemia in her daughter, she was suspected of hematological system diseases like leukemia. Infection was also considered because of the physical examination and relevant laboratory abnormalities. Before and after admission to our hospital, case 1 was administered different antibiotics for anti-infection, but the fever had not been remitted. In addition, no sufficient evidence was found to show a myeloid blood disease. Worse still, no pathogen was seen in the bone marrow aspiration smear. Until the *Leishmania* reported by mNGS detection, it was also found in the bone marrow aspiration smear after repeated and careful examinations.

Case 2 suffered from underdiagnosis severely, who had been hospitalized in different hospitals for nearly 1 month and spent massively. Before admitting to our hospital, he had been administered various antibiotics, such as piperacillin sodium/tazobactam, but without any improvement. He was suspected of infection or hemophagocytic syndrome after admission to our hospital. But no blood or respiratory tract pathogenic microorganism was detected. Within 14 days, the bone marrow aspiration smear was performed three times but showed no abnormality. On day 16, peripheral blood mNGS was arranged, and *Leishmania* genus- and *L. donovani*-specific sequences were reported. According to the result of mNGS, we re-examined the bone marrow smear and found ten suspected *Leishmania*, which was confirmed by the positive outcome of the rK39 antibody test. Finally, he was diagnosed with visceral leishmaniasis and hemophagocytic syndrome, was treated timely, and discharged from the hospital with drugs, and the outcome was so satisfactory. Then, analysis of gene mutation in hemophagocytic syndrome found no disease-causing mutation. Therefore, hemophagocytic syndrome is more likely to be associated with the infection of *Leishmania*.

Furthermore, mNGS is available for *Leishmania* detection not only in bone marrow aspirate (13, 14), especially for quick and accurate diagnosis in patients with suspected leishmaniasis (14, 15), but also in the easy-to-obtain peripheral blood. Sequencing readings of *L. infantum* and *L. donovani* also have been identified by mNGS using peripheral blood (16). During the continuous reproduction process of *Leishmania* in the blood circulation system, the nucleic acid released into the peripheral blood forms mcfDNA and mcfRNA along with apoptosis occurring, which is the basis for the detection of *Leishmania* nucleic acid in the peripheral blood by mNGS (17). Even if the pathogen has been killed, its mcfDNA and mcfRNA may still exist in the peripheral blood for a while, and mcfRNA will be degraded faster because of its shorter half-life. Therefore, mcfRNA is superior to mcfDNA in monitoring the number of reads to evaluate the treatment efficacy of sodium stibogluconate, which is commonly used to inhibit *L. donovani* in the treatment of visceral leishmaniasis (18). Our mNGS reports indicated a marked decrease in the reads number of the *Leishmania*-specific sequence in the two patients' peripheral blood after a period of sodium stibogluconate treatment.

In this study, we proved the feasibility of mNGS in detecting causative pathogens and diagnosing visceral leishmaniasis from peripheral blood samples, dramatically avoiding misdiagnosis or underdiagnosis. Changes in the number of reads of mcfDNA and mcfRNA sequences yielded in mNGS can be taken as evidence of effective anthelmintic therapy. Finally, the relatively high cost of mNGS is the main shortcoming that limits its sample size and hinders its wide use in clinical. Once this challenge is addressed, further studies with a more extensive sampling size are more likely to validate *Leishmania* detection's reliability *via* mNGS in blood samples.

## Data availability statement

The datasets presented in this article are not readily available because of ethical/privacy restrictions. Requests to access the datasets should be directed to the corresponding author.

## Ethics statement

Written informed consent was obtained from the individual(s) for the publication of any potentially identifiable images or data included in this article.

## Author contributions

QL and XL performed the study concept and design, manuscript review, and revision. FC and DH contributed to the data acquisition and analysis. YF and LT performed development and methodology and writing. XT and JM provided data acquisition, analysis, and interpretation. All authors contributed to the article and approved the submitted version.

## Supplementary material

Supplementary Figure 1 A macrophage infected with amastigotes in leishmaniasis case 1. Bone marrow aspiration shows the *Leishmania* spp amastigotes (arrow) in case 1. Original magnification ×50.

Supplementary Figure 2 A macrophage infected with amastigotes in leishmaniasis case 2. Bone marrow aspiration shows the *Leishmania* spp amastigotes (arrow) in case 2. Original magnification ×40.
