# Supplementary figures and images for: Case report: Application of metagenomic next-generation sequencing in the diagnosis of visceral leishmaniasis and its treatment evaluation
Source: Front Med (Lausanne). 2023 Jan 13;9:1044043. doi: 10.3389/fmed.2022.1044043 (PMC9880153; doi:10.3389/fmed.2022.1044043)

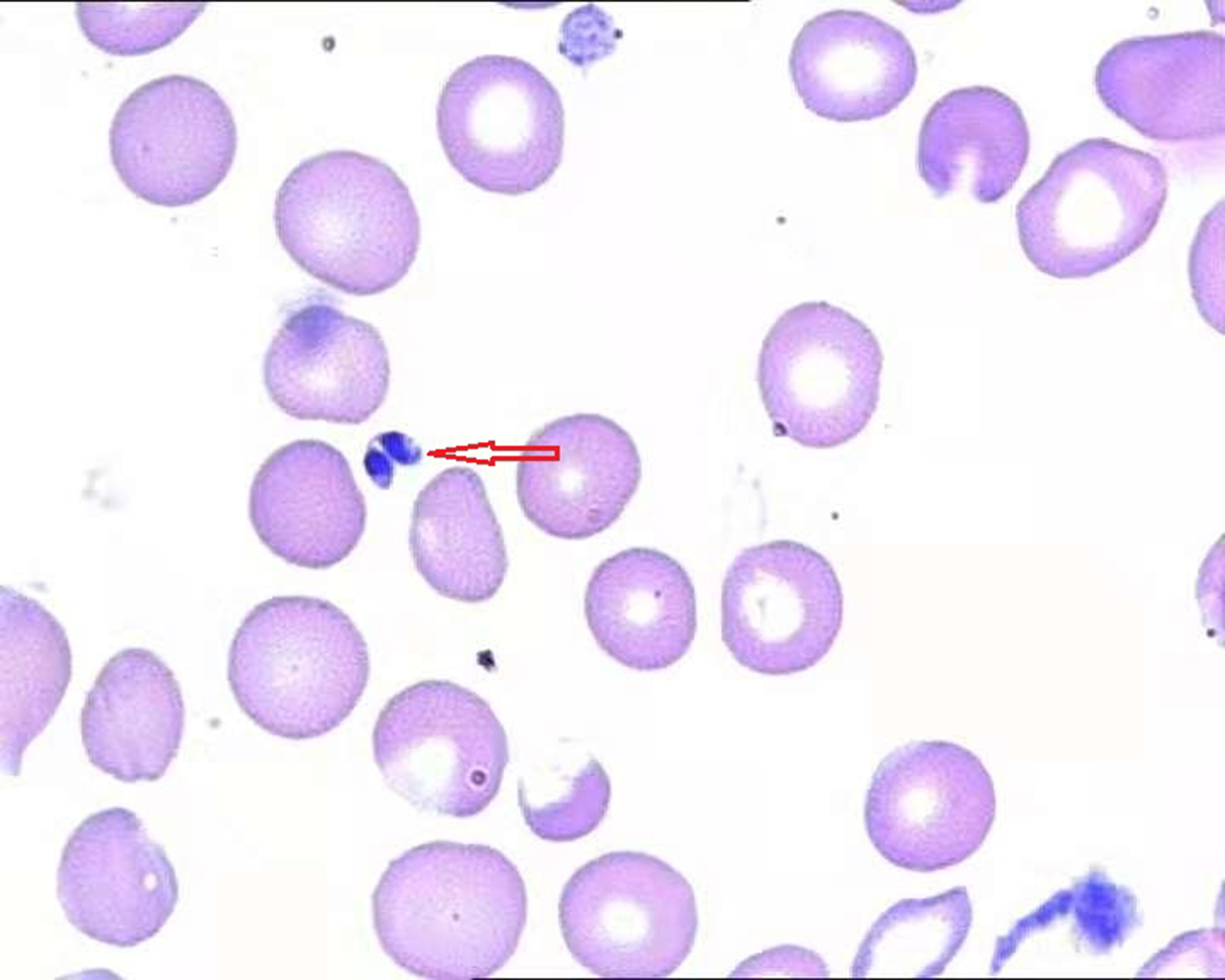

Supplement: Supplementary Figure 1 — A macrophage infected with amastigotes in leishmaniasis case 1. Bone marrow aspiration shows the Leishmania spp amastigotes (arrow) in case 1. Original magnification ×50. [file Image_1.JPEG]

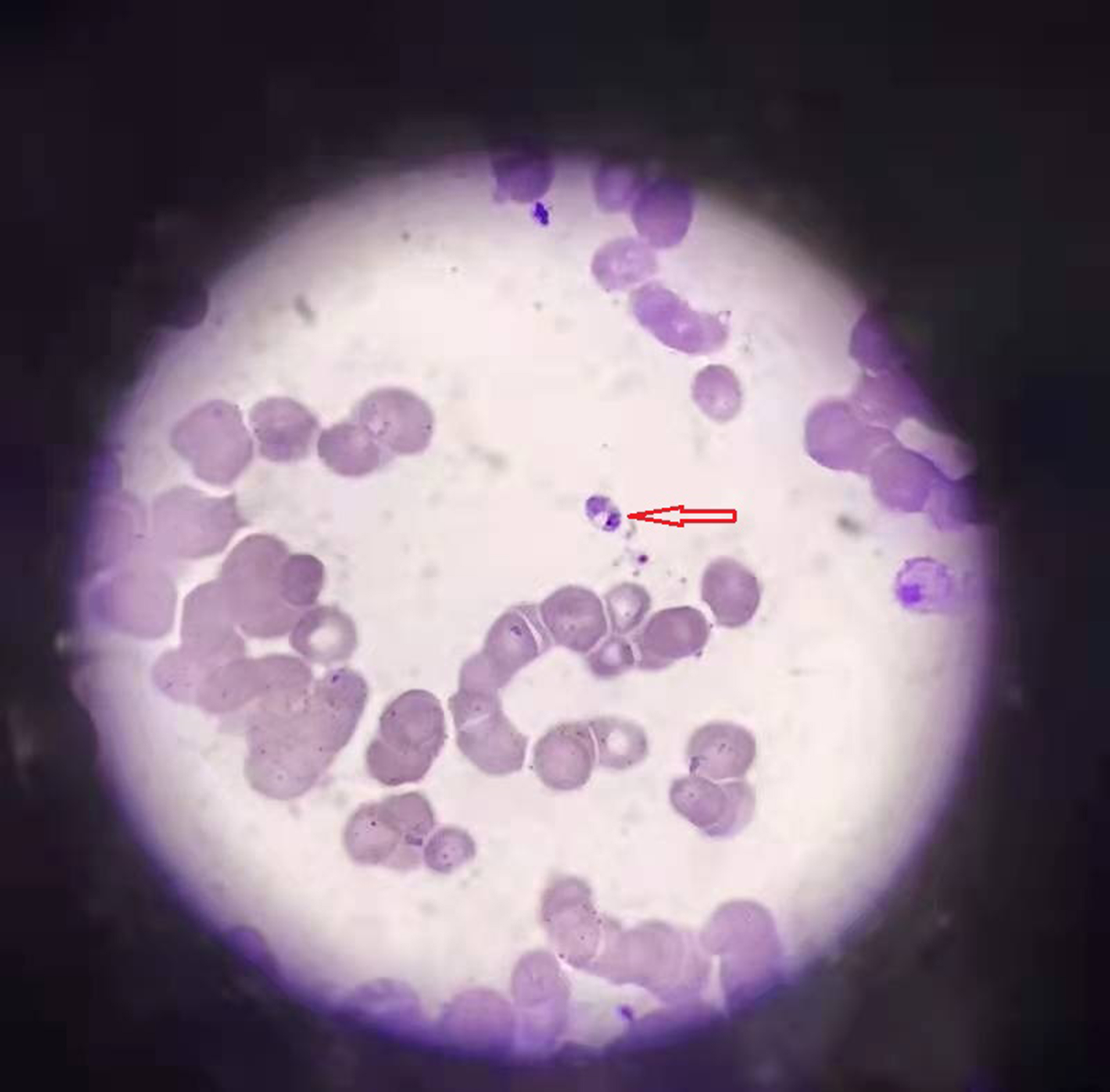

Supplement: Supplementary Figure 2 — A macrophage infected with amastigotes in leishmaniasis case 2. Bone marrow aspiration shows the Leishmania spp amastigotes (arrow) in case 2. Original magnification ×40. [file Image_2.JPEG]
